# Small-extracellular vesicles and their microRNA cargo from porcine follicular fluids: the potential association with oocyte quality

**DOI:** 10.1186/s40104-022-00723-1

**Published:** 2022-06-20

**Authors:** Ahmed Gad, Matej Murin, Alexandra Bartkova, Veronika Kinterova, Katerina Marcollova, Jozef Laurincik, Radek Prochazka

**Affiliations:** 1 Institute of Animal Physiology and Genetics, Czech Academy of Sciences, 27721 Liběchov, Czech Republic; 2 Department of Animal Production, Faculty of Agriculture, Cairo University, Giza, 12613 Egypt; 3 Department of Botany and Genetics, Faculty of Natural Sciences, Constantine the Philosopher University in Nitra, 94901 Nitra, Slovakia

**Keywords:** Extracellular vesicles, Follicular fluids, MiRNA, Oocyte quality, Porcine

## Abstract

**Background:**

Ovarian follicular fluids (FFs) contain several kinds of regulatory factors that maintain a suitable microenvironment for oocyte development. Extracellular vesicles (EVs) are among the factors that play essential roles in regulating follicle and oocyte development through their cargo molecules that include microRNAs (miRNAs). This study aimed to investigate small-EV (s-EV) miRNAs in porcine FFs and their potential association with oocyte quality.

**Methods:**

Individual aspirated oocytes were stained with lissamine green B stain (LB), a vital stain for oocyte quality, and each oocyte was classified as high-quality (unstained; HQ) or low-quality (stained; LQ). FFs corresponding to oocytes were pooled together into HQ and LQ groups. Small-EVs were isolated from FFs, characterized, and their miRNA cargo was identified using the Illumina NovaSeq sequencing platform. Additionally, s-EVs from the HQ and LQ groups were utilized to investigate their effect on oocyte development after co-incubation during in vitro maturation.

**Results:**

A total of 19 miRNAs (including miR-125b, miR-193a-5p, and miR-320) were significantly upregulated, while 23 (including miR-9, miR-206, and miR-6516) were downregulated in the HQ compared to the LQ group. Apoptosis, p53 signaling, and cAMP signaling were among the top pathways targeted by the elevated miRNAs in the HQ group while oocyte meiosis, gap junction, and TGF-beta signaling were among the top pathways targeted by the elevated miRNAs in the LQ group. The supplementation of small-EVs during maturation does not affect the oocyte developmental rates. However, LQ s-EVs increase the proportion of oocytes with homogeneous mitochondrial distribution and decrease the proportion of heterogeneous distribution.

**Conclusion:**

Our findings indicated that FF-EVs contain different miRNA cargos associated with oocyte quality and could affect the mitochondrial distribution patterns during oocyte maturation.

**Supplementary Information:**

The online version contains supplementary material available at 10.1186/s40104-022-00723-1.

## Background

Oocyte developmental competence is commonly defined as the ability of the oocyte to mature, fertilize, and develop to the blastocyst stage. Several factors, including oocyte quality, could influence this ability and subsequently determine developmental competence. Various methods including morphological, biochemical, and molecular techniques, are being used to assess oocyte quality [1], with the aim of enhancing the efficiency of assisted reproductive technologies (ARTs). At the molecular level, several studies have been done to determine molecular markers from follicular cells surrounding the oocyte or follicular fluids (FFs) as a non-invasive method that predicts oocyte quality [2]. Ovarian FFs contain several kinds of regulatory factors that maintain a suitable microenvironment for oocyte development and follicular intercellular communication [3]. This kind of communication within the follicle is essential and could determine oocyte quality and consequently developmental competence [4]. One of the recently discovered mechanisms that facilitate and modulate intercellular crosstalk is via the extracellular vesicles (EVs). EVs, including exosomes and microvesicles, are cell-derived lipid bilayer membranous particles that contain different biomolecules, including proteins, lipids, RNAs, and microRNAs (miRNAs). These particles are secreted by almost all cell types and can be found in all body fluids [5]. The ability of EVs to modulate intercellular crosstalk is through the capacity to transfer their biomolecular cargos between different cells after being secreted in body fluids [5]. After this discovery, several studies revealed the fundamental roles of EVs in the different reproduction processes in domestic animals (reviewed by Llobat [6]) and the subsequent improvement of ART outcomes [7].

MiRNAs, a small non-coding RNA class, are posttranscriptional regulators of gene expression by binding specific mRNA target sequences for degradation or translational repression [8]. Extracellular miRNAs were discovered in various body fluids in a stable form, which supports their potential utilization as non-invasive biomarkers for several physiopathological conditions [9]. The availability and stability of extracellular miRNAs within body fluids are supported by either the incorporation of miRNAs within the EVs or by binding with specific protein complexes [10]. In 2007, miRNAs were identified for the first time within cell-secreted EVs that can be delivered and regulate several functions in the target cells [11]. In ovarian FFs, it has been reported that the majority of miRNAs are found within EVs [12] and are suggested to play a role in follicle development and other ovarian functions [13]. Consequently, alterations in the FF miRNAs could reflect the status of the oocyte quality and its developmental competence [14]. Therefore, the objectives of this study were to identify small-EV (s-EV) miRNA differences in porcine FFs in association with oocyte quality and to investigate the effect of s-EVs on oocyte developmental competence. The identified miRNAs could be used as non-invasive biomarkers for oocyte selection. Moreover, our results provided more insights into the potential role of FF-EVs during oocyte maturation.

## Materials and methods

### Chemicals and supplements

All plastic materials and chemicals were purchased from Thermo Fisher Scientific (Waltham, MA, USA) and Merck (Kenilworth, NJ, USA), respectively, unless stated otherwise. All media were prepared fresh and sterilized using 0.22 μm syringe filters.

### Collection of follicular fluids, COCs staining, and classification

**Fig. 1:**
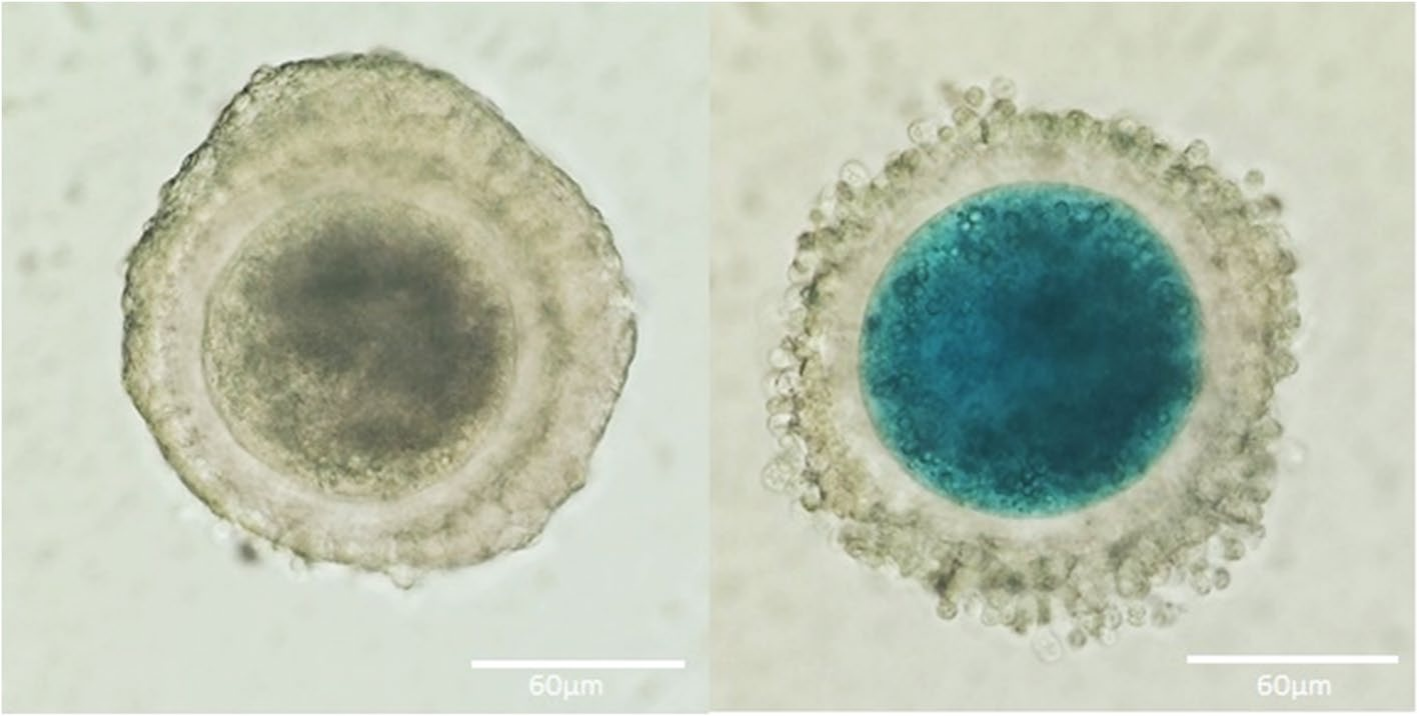
Classification of porcine COCs after lissamine green B (LB) staining. Left: Unstained COC (high-quality; HQ). Right: Stained COC (low-quality; LQ). Scale bar, 60 μm

Porcine ovaries of prepubertal gilts (Landrace × Large White, 6–8 months of age, 90–120 kg) were collected from a local slaughterhouse and transported to the lab in a thermos flask within 2 h. Ovaries were washed three times with saline solution. Follicular fluids and COCs were aspirated from individual healthy follicles (3–6 mm in diameter, measured as previously recommended [15]) using a 25-gauge needle attached to a 1-mL syringe (Braun, Germany). Each COC and its corresponding FF were allocated in separate wells in a 96-well plate. COCs were washed once in PXM-HEPES (HEPES buffered porcine X medium [16]) and then stained for 15 min at room temperature with 0.5% lissamine green B stain (LB), a vital synthetic stain for determining oocyte quality and competence [17, 18]. Each COC was classified separately according to the oocyte stain into high-quality (unstained; HQ) and low-quality (stained; LQ) as presented in Fig. 1. FFs corresponding to oocytes were pooled together into HQ and LQ groups and used for the isolation of small-extracellular vesicles (s-EVs).

### Isolation of small-extracellular vesicles from follicular fluid

To isolate s-EVs (< 200 nm), pooled FFs (~ 1 mL/replicate) from each group were centrifuged at 700 × *g* for 10 min to pellet cells, at 2000 × *g* for 10 min to remove cell debris, and at 12,000 × *g* for 30 min to remove large particles and protein aggregates. All centrifugation steps were performed at 4 °C. The remaining supernatants were filtrated through a 0.2-μm syringe filter to eliminate larger vesicles. Small-EVs were isolated from 0.5 mL filtered FFs (3 replicates/group) using an Exo-spin kit (Cell Guidance Systems, UK) and eluted in PBS according to the manufacturer’s protocol. For electron microscope imaging and western blot analysis, samples were kept at 4 °C until analysed. The remaining samples were stored at − 80 °C for further analysis. All relevant data regarding s-EV isolation and characterization were submitted to the EV-TRACK knowledgebase [19] with the EV-TRACK ID EV210251.

### Nanoparticle tracking analysis (NTA)

To determine vesicle size and concentration, samples were diluted in PBS (1:200) and NTA was conducted in a ZetaView instrument (Particle Metrix, Germany) in scatter mode (488 nm laser). Measurements were performed in two cycles by scanning 11 cell positions each, and the videos were analysed using the software ZetaView (version 8.05.12).

### Transmission electron microscopy (TEM)

For morphological evaluation, isolated s-EV samples were placed into formvar/carbon-coated 400 mesh copper grids for 20 min at room temperature. Then, the grids were incubated with 2% formaldehyde in PBS for 20 min, followed by 6 washes for 2 min each with ultrapure water. The grids were stained with uranyl acetate (2%) for 12 min, washed in water, left to dry, and then examined in a Jeol JEM-1400 FLASH transmission electron microscope (Tokyo, Japan) equipped with Matataki 2k×2k CMOS camera at 80 kV.

### Western blot analysis

Samples of s-EV, filtered follicular fluid, or follicular cells containing 40 μg of total protein were mixed with Laemmli buffer containing 2% sodium dodecyl sulphate (SDS) and 5% 2-mercaptoethanol. Samples were boiled at 100 °C for 3 min and stored frozen at − 20 °C. Subsequently, proteins were separated in 10% or 12% acrylamide/SDS gels and transferred to Immobilon-P membranes (Millipore, Bedford, MA, USA). Membranes were blocked in 5% low-fat dry milk in Tris-buffered saline (TBS) with 0.5% Tween 20 for 2 h at room temperature and then incubated with a primary antibody at 4 °C overnight. The primary antibodies were anti-CD63 (Abcam, Ab 118307, diluted 1:1000 in 2% BSA in TBS-Tween), anti-Alix (Abcam, Ab 88388, diluted 1:500 in 5% milk), and anti-TSG101 (Santa Cruz Biotechnology, Sc-7964, diluted in 1:200 in 5% milk) raised against exosome-specific markers, and anti-Cytochrome C (Abcam, Ab 90529, diluted 1:1000 in 2% BSA) and anti-ATP5A (Abcam, Ab14748, diluted 1:2000 in 5% milk) raised against somatic cell-specific markers. The secondary antibodies (Amersham ECL anti-mouse or anti-rabbit IgG, GE Healthcare, Little Chalfont, UK) were diluted 1:5000 in 2% BSA in TBS-Tween. The membranes were incubated with the secondary antibody for 1 h at room temperature and then washed intensively in TBS-Tween. The immune reactions were detected by enhanced chemiluminescence (Pierce, Rockford, IL, USA) according to the manufacturer’s instructions and captured on CL-XPosure film (ThermoFisher Scientific).

### Total RNA extraction, library preparation, and sequencing

Total RNA, including miRNA was isolated from EV samples using a miRNeasy Micro Kit (Qiagen, Hilden, Germany) that combines the phenol/guanidine-based lysis of samples and silica membrane-based purification of total RNA, according to the manufacturer’s instructions. The RNA concentration and size distribution were analyzed using an Agilent RNA 6000 Pico kit in an Agilent 2100 Bioanalyzer (Agilent Technologies, Santa Clara, CA, USA). Small-RNA libraries were prepared for next-generation sequencing (NGS) using a QIAseq miRNA Library Kit (Qiagen) according to the manufacturer’s instructions. Library quantity and quality assessments were performed using a Qubit DNA HS Assay Kit in a Qubit 4 Fluorometer (Thermo Fisher Scientific) and Agilent DNA High Sensitivity kit in an Agilent 2100 Bioanalyzer (Agilent Technologies), respectively. The libraries were pooled in equimolar ratios and then sequenced in a NovaSeq6000 sequencing instrument (Illumina, Inc., San Diego, CA, USA) as single-end reads.

### Sequencing data analysis

FASTQ files were generated for each sample using the software bcl2fastq (Illumina Inc., San Diego, CA), and their quality was checked using the FastQC tool version 0.11.9. Data were analyzed using the software CLC Genomics Workbench, version 21 (www.qiagenbioinformatics.com). Raw sequencing reads were trimmed based on quality score (Q-score > 30), ambiguous nucleotides (maximum two nucleotides allowed), read length (≥15 nucleotides) and removing adapter sequences. Reads were mapped to the porcine (*Sus scrofa*) reference genome (Sscrofa11.1) and annotated against porcine precursor and mature miRNAs listed in the mirBase database (release 22) using the CLC Genomics Workbench RNA-Seq Analysis and Quantify miRNA tools, respectively, applying the default software parameters. Raw expression data were normalized using the trimmed mean of M-values normalization method (TMM normalization) [20] and presented as TMM-adjusted Counts Per Million (CPM). The CLC Genomics Workbench Differential Expression tool was used for the expression analysis comparison of the two groups. MiRNAs with fold change (FC) ≥ 2, *P*-adjusted value (FDR [21]) < 0.05, and CPM > 5 in the enriched group were considered differentially expressed (DE). The raw FASTQ files and processed CSV files have been deposited in the NCBI’s Gene Expression Omnibus (GEO) with the accession number GSE181182.

### Target gene prediction and ontological classification

Genes targeted by DE-miRNA were identified using the miRWalk database [22]. Within the miRWalk, validated target genes from miRTarBase (version 7.0) and commonly target genes predicted by TargetScan (version 7.1) and miRDB (release 5.0) were selected for ontological classification and pathway analysis using the DAVID bioinformatics web tool (https://david.abcc.ncifcrf.gov/). Pathways were determined from the KEGG database [23], and interaction networks of the targeted genes and the identified pathways were constructed with Cytoscape [24] and its plug-in ClueGO [25].

### DE-miRNA validation using droplet digital PCR (ddPCR)

To validate the miRNA-seq data, we performed a PCR analysis for a selected group of 11 DE-miRNAs. The absolute copy numbers of the selected DE-miRNAs were quantified in the EVs samples using specific TaqMan miRNA Assays (Applied Biosystems, Foster City, CA, USA) in a ddPCR system (Bio-Rad Inc., Hemel Hempstead, UK) according to the manufacturer’s instructions and as previously described [26]. The copy numbers of the selected DE-miRNAs were normalized to miR-26b-5p, the most stably expressed miRNA across all samples, according to the analysis with the software NormFinder.

### Co-incubation of s-EVs with COCs during maturation

COCs were collected as mentioned above using a 20-gauge needle attached to a 10-mL syringe and then morphologically evaluated under a stereomicroscope (Zeiss Stemi 508, magnification × 50). Only those with at least three layers of cumulus cells and an evenly granulated ooplasm were used. COCs were washed twice in the maturation medium (Medium 199) supplemented with 0.005% gentamicin (Roth 0233), 0.0022% sodium pyruvate, 0.01% L-glutamine, 0.1% BSA, 10 ng/mL EGF, 40 ng/mL FGF2, 20 ng/mL IGF1, 2000 IU/mL LIF, 0.57 mmol/L L-Cysteine, 10 IU/mL PMSG and 10 IU/mL HCG. COCs were cultivated in 4-well dishes (30–50 per well) for 44 h at 38.5 °C under a 5% CO_2_ atmosphere in 500 μL maturation medium supplemented with or without (control) s-EV particles (~ 200 million particles/mL) isolated from HQ or LQ FF groups. COCs cultivated in the same maturation media supplemented with a volume of PBS similar to the s-EVs were used as the negative control group (NC).

### Parthenogenetic activation and embryo cultivation

After maturation, cumulus cells were removed from COCs by pipetting and washed twice in PXM-HEPES. Oocytes were activated using 10 μmol/L ionomycin in PXM-HEPES for 5 min, washed twice in porcine zygote medium 3 (PZM 3) [27] supplemented with 2 mmol/L 6-dimethylaminopurine, and cultivated for 5 h at 38.5 °C under a 5% CO_2_ atmosphere. A group of 30–50 putative parthenotes was washed twice in PZM 3 and cultivated for 7 d in 4-well dishes in 500 μL of PZM 3 medium at 38.5 °C under a 5% CO_2_ atmosphere. The cleavage rate was assessed after 40 h, and the ability of the embryos to reach the blastocyst stage as well as the number of nuclei in blastocysts were analyzed after 168 h of cultivation. Blastocysts were fixed using 4% paraformaldehyde and mounted on glass slides using DAPI mounting medium. Each blastocyst was scanned using a confocal microscope (Leica SP5, Germany), and the number of nuclei was counted using the software ImageJ.

### Oocyte mitochondrial activity and distribution patterns

Matured COCs were denuded as described above. Oocytes were washed three times in PBS, stained with 300 nmol/L Mitotracker Orange kit (Thermo Fisher Scientific, USA) for 30 min in the dark at 38.5 °C. Then, oocytes were washed three times in PBS at 38.5 °C, fixed for 15 min in 4% paraformaldehyde, and mounted on glass slides using DAPI mounting medium. Oocytes were scanned in a confocal microscope (Leica SP5, Germany) and mitochondrial activity was analyzed using the software ImageJ. The distribution patterns of mitochondria were characterized by observing (at 630-fold magnification) labeled mitochondria with an active oxidative state. We classified the distribution and aggregation patterns mainly as homogeneous, heterogeneous, and clustered distribution according to the previously reported classification [28, 29].

### Statistical analysis

All experiments were done in at least three replicates. Data normality was checked using the Shapiro–Wilk test. Maturation and developmental rates and blastocyst nuclei numbers were analyzed using one-way ANOVA (SigmaPlot 12, US) followed by Tukey’s test to detect differences between the means and expressed as mean ± SD. Categorical variables were identified using the chi-square test. PCR data were statistically analyzed using Student’s *t*-test. The statistical significance level was defined at *P* < 0.05.

## Results

### Characterization of s-EVs from porcine follicular fluids

**Fig. 2:**
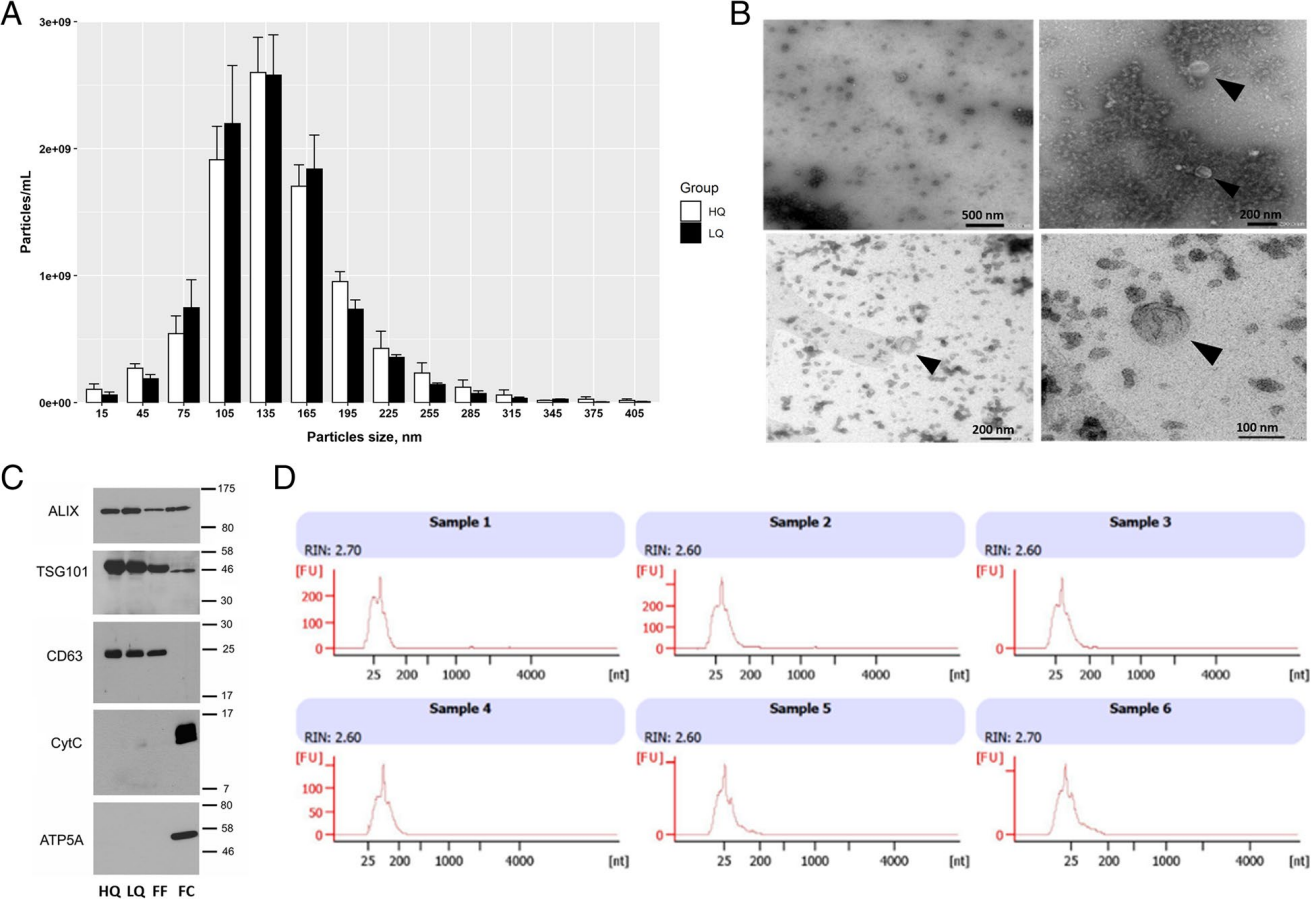
Morphological and molecular characterization of s-EVs. **A** The concentration and size distribution of s-EVs isolated from follicular fluids (FFs) corresponding to high- (HQ) or low-quality (LQ) oocytes analysed by NTA. **B** Transmission electron microscopy (TEM) representative photos of isolated s-EVs showing the lipid bilayer membrane and the cup-shaped EV particles (black arrows). Scale bar of 500 and 200 nm for the upper left and right photos, respectively, and of 200 and 100 nm for the lower left and right photos, respectively). **C** Immunoblotting analysis of EV-specific protein markers (ALIX, TSG101, and CD63) and cellular specific protein markers (CytC and ATP5A) in the HQ and LQ EV groups, as well as in filtered FF and follicular cell lysate (FC) as positive and negative controls, respectively. Scale numbers are in kDa. **D** RNA size distribution from s-EV samples of HQ (sample 1–3) and LQ (sample 4–6) groups analysed with a bioanalyzer (Agilent Technologies)

Different morphological and molecular analyses were done to determine the characteristics and the purity of s-EV isolated samples. Vesicle size and concentration were determined in each sample using NTA. The concentration of s-EVs from the HQ and LQ FF groups was 8.98E+ 09 ± 3.43E+ 08 and 8.97E+ 09 ± 1.25E+ 09 particles/mL, the median size was 135.7 ± 5.3 and 132.6 ± 3.3 nm, respectively, and the mode was 135 nm in both groups, with no significant differences between them (Fig. 2A). Imaging with TEM revealed the presence of s-EVs with visible lipid bilayer membranes (Fig. 2B). Western blot analysis identified the presence of EV-specific markers (ALIX, TSG101, and CD63) in both s-EV groups and the filtered FFs. Additionally, cellular specific protein markers (CytC and ATP5A) were only detected in the cell lysates, but not in the isolated s-EVs, indicating the absence of other cellular membrane contamination in the EV samples (Fig. 2C). Lastly, the size distribution of the total RNA isolated from s-EV samples exhibited clear peaks for small-RNA and the absence of ribosomal-RNA peaks, confirming the samples to be free of cells and cellular components (Fig. 2D).

### MiRNA expression profiles of s-EVs isolated from follicular fluids

**Fig. 3:**
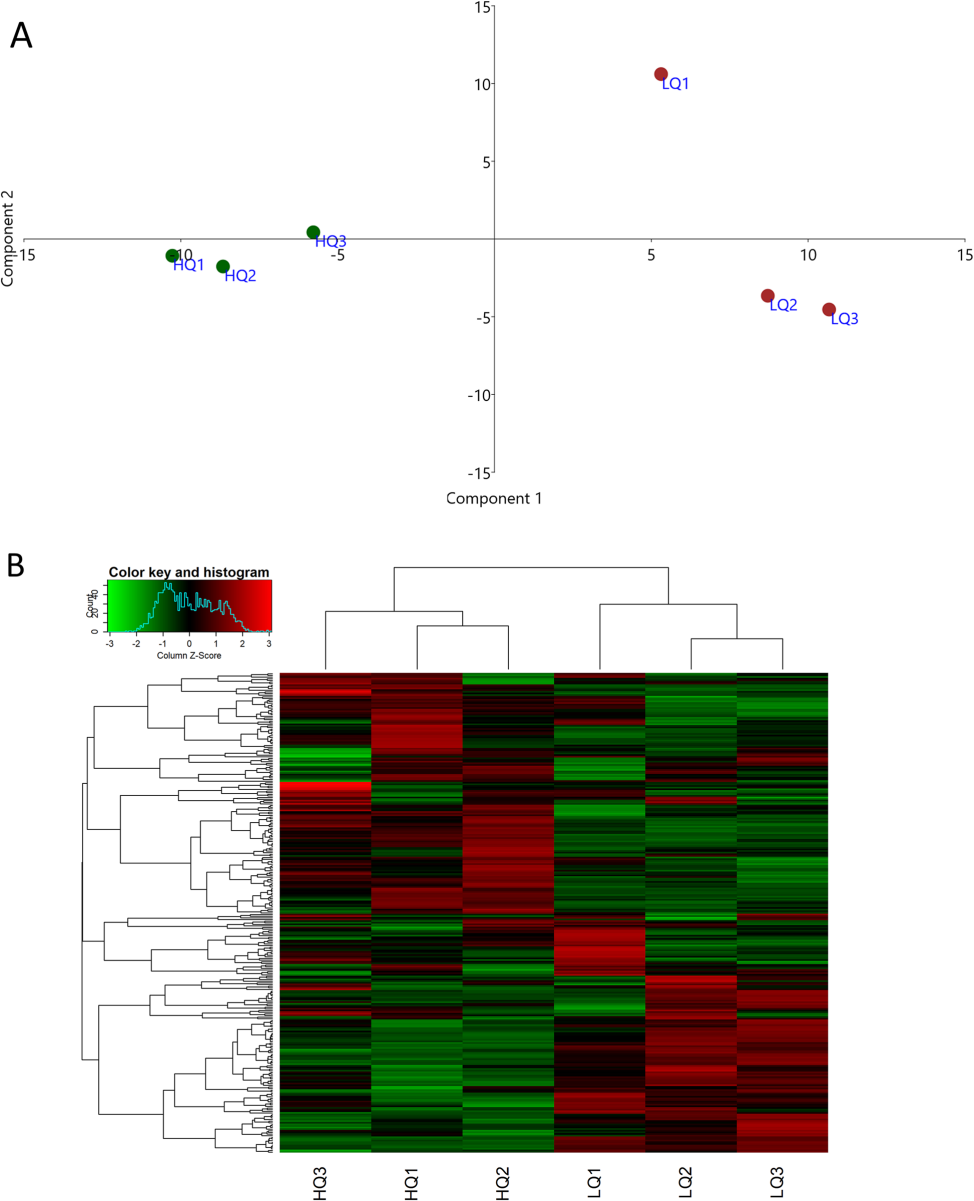
Samples clustering. **A** Principal component analysis (PCA). **B** Heatmap and hierarchical clustering. HQ1-HQ3: high-quality s-EVs replicates, LQ1-LQ3: low-quality s-EVs replicates

**Fig. 4:**
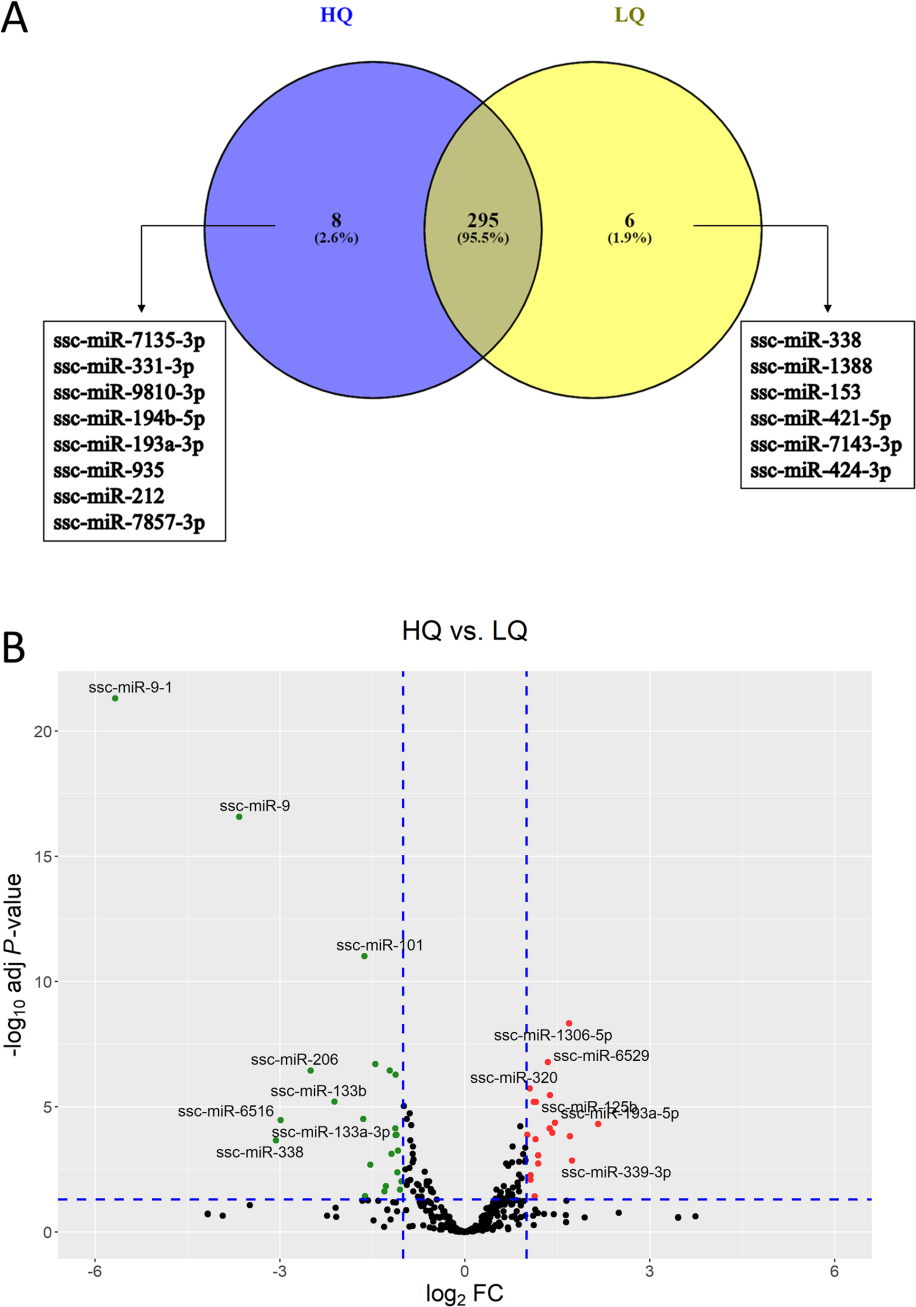
Differential expression analysis. **A** Venn diagram for commonly and exclusively expressed miRNAs in HQ and LQ s-EVs groups. **B** Volcano plot of expressed miRNAs. Up- and downregulated miRNAs in the HQ compared to the LQ s-EVs groups are labeled with red and green points, respectively

Small-RNA libraries were prepared from six different EV samples (3 replicates per group) to identify the expressed miRNAs in each. RNA-seq analysis gave an average number of 27 million raw reads per library with an average of 16 million reads being retained after trimming and quality control. An average of 89% of reads were mapped to the porcine genome, and the average proportion of annotated miRNAs was 15% (Additional file 1: Table S1). Various types of small non-coding RNA (sncRNA), including misc_RNA, snRNA, and snoRNA were also identified in all samples. However, the majority of sncRNA reads were assigned to miRNAs (Additional file 2: Fig. S1). Heatmap clustering and principal component analysis (PCA) were performed on the miRNA CPM values. The results showed a clear clustering of the three biological replicates of each group, with a clear separation between the two groups. The first two components of the PCA explained around 68.5% of the existing variances (Fig. 3). A miRNA with an average value of CPM > 1 was considered to be expressed. Accordingly, a total of 303 and 301 miRNAs were expressed in the HQ and LQ groups, respectively, with 295 miRNAs being expressed in both groups (Fig. 4A). A complete list of all expressed miRNAs is presented in Additional file 1: Table S2, and the top 20 most abundant miRNAs in each group are presented in Table 1. Interestingly, miR-27b-3p, miR-140-3p, miR-29a-3p, miR-202-5p, and miR-16 were the top 5 expressed miRNAs in both groups, in which they accounted for 42% and 49.2% of the miRNAs sequence reads in the HQ and LQ groups, respectively (Table 1).

**Table 1:** List of top 20 most abundant miRNAs in small-extracellular vesicles obtained from follicular fluids of high (HQ) or low (LQ) quality corresponding oocytes

miRNA	HQ group CPM	%^**a**^	miRNA	LQ group CPM	%^**a**^
ssc-miR-27b-3p	102,691.3	10.9	ssc-miR-27b-3p	155,625.9	14.8
ssc-miR-140-3p	80,017.22	8.6	ssc-miR-16	115,577.2	11.4
ssc-miR-29a-3p	78,734.08	8.3	ssc-miR-29a-3p	99,770.91	9.2
ssc-miR-202-5p	71,447.84	7.5	ssc-miR-140-3p	82,856.62	7.4
ssc-miR-16	62,302.65	6.7	ssc-miR-202-5p	70,526.08	6.4
ssc-let-7c	39,828.22	4.3	ssc-miR-29c	51,664.11	4.7
ssc-miR-423-5p	32,738.73	3.6	ssc-miR-152	44,794.39	4.3
ssc-miR-128	31,129.2	3.4	ssc-let-7c	30,586.53	2.5
ssc-miR-152	28,718.56	3.1	ssc-miR-146a-5p	26,660.02	2.5
ssc-miR-29c	28,297.13	3.0	ssc-let-7i-5p	26,275.77	2.5
ssc-let-7a	26,486.27	2.9	ssc-miR-676-3p	22,826.69	2.2
ssc-miR-676-3p	24,661.24	2.7	ssc-miR-30e-5p	22,798.43	2.2
ssc-miR-146a-5p	23,950.27	2.6	ssc-miR-128	22,443.82	2.2
ssc-miR-24-3p	21,119.86	2.3	ssc-let-7a	16,416.26	1.4
ssc-miR-10b	19,325.57	2.1	ssc-miR-24-3p	16,005.13	1.5
ssc-miR-191	17,564.45	1.9	ssc-miR-30a-5p	15,408.66	1.4
ssc-let-7f-5p	17,071.41	1.8	ssc-miR-423-5p	14,893.75	1.3
ssc-let-7i-5p	16,857.99	1.8	ssc-miR-10b	13,573.69	1.1
ssc-miR-30e-5p	12,606.02	1.3	ssc-let-7f-5p	12,654.58	1.1
ssc-miR-30a-5p	11,671.15	1.2	ssc-miR-19b	11,413.06	1.2

*CPM* average Counts Per Million mapped reads

^a^Percentage of the miRNAs sequence reads

### Differentially expressed miRNAs and ontological classification

Differential expression analysis of miRNAs revealed that 19 miRNAs (including miR-193a-5p, miR-339-3p, ssc-miR-132, ssc-miR-125b and ssc-miR-320) and 23 miRNAs (including miR-9-1, miR-9, miR-206, miR-133b, and miR-133a-3p) were significantly up- and downregulated (FC ≥ 2, FDR < 0.05, and CPM > 5 in the enriched group) in the HQ compared to the LQ s-EVs group, respectively (Fig. 4B, Table 2).

**Table 2:** Differentially expressed (DE) miRNAs in small-extracellular vesicles obtained from follicular fluids of high (HQ) compared to low (LQ) quality corresponding oocytes

Upregulated miRNAs	FC	FDR	Downregulated miRNAs	FC	FDR
ssc-miR-193a-5p	4.48	4.83E-05	ssc-miR-9-1	−51.16	4.90E-22
ssc-miR-339-3p	3.34	1.38E-03	ssc-miR-9	−12.67	2.60E-17
ssc-miR-671-3p	3.26	1.48E-04	ssc-miR-338	−8.39	2.15E-04
ssc-miR-1306-5p	3.22	4.63E-09	ssc-miR-6516	−7.95	3.34E-05
ssc-miR-885-5p	2.75	4.28E-05	ssc-miR-206	−5.67	3.51E-07
ssc-miR-7142-3p	2.67	1.07E-04	ssc-miR-133b	−4.34	6.23E-06
ssc-miR-125b	2.60	3.40E-06	ssc-miR-133a-3p	−3.14	3.04E-05
ssc-miR-132	2.60	7.14E-05	ssc-miR-101	−3.10	9.57E-12
ssc-miR-6529	2.54	1.62E-07	ssc-miR-219b-3p	−3.07	3.67E-02
ssc-miR-296-5p	2.28	1.82E-03	ssc-miR-143-5p	−2.90	2.04E-03
ssc-miR-125a	2.27	8.51E-04	ssc-miR-142-5p	−2.74	1.94E-07
ssc-miR-423-3p	2.22	6.35E-06	ssc-miR-451	−2.48	2.34E-02
ssc-miR-423-5p	2.21	1.92E-04	ssc-miR-10,388	−2.43	1.44E-02
ssc-miR-7144-5p	2.19	3.78E-02	ssc-miR-199b-5p	−2.33	3.51E-07
ssc-let-7d-3p	2.17	6.33E-06	ssc-miR-20a-5p	−2.29	7.58E-04
ssc-miR-551a	2.09	5.36E-03	ssc-miR-19a	−2.19	7.14E-05
ssc-miR-149	2.09	7.98E-03	ssc-miR-301	−2.18	1.34E-04
ssc-miR-320	2.07	1.85E-06	ssc-miR-126-5p	−2.18	5.10E-07
ssc-miR-425-5p	2.02	1.29E-04	ssc-miR-218b	−2.16	1.29E-04
			ssc-miR-424-5p	−2.14	4.15E-03
			ssc-miR-190b	−2.13	5.58E-04
			ssc-miR-545-5p	−2.08	2.04E-02
			ssc-miR-95	−2.04	9.49E-03

*FC* Fold Change, *FDR* False Discovery Rate

**Fig. 5:**
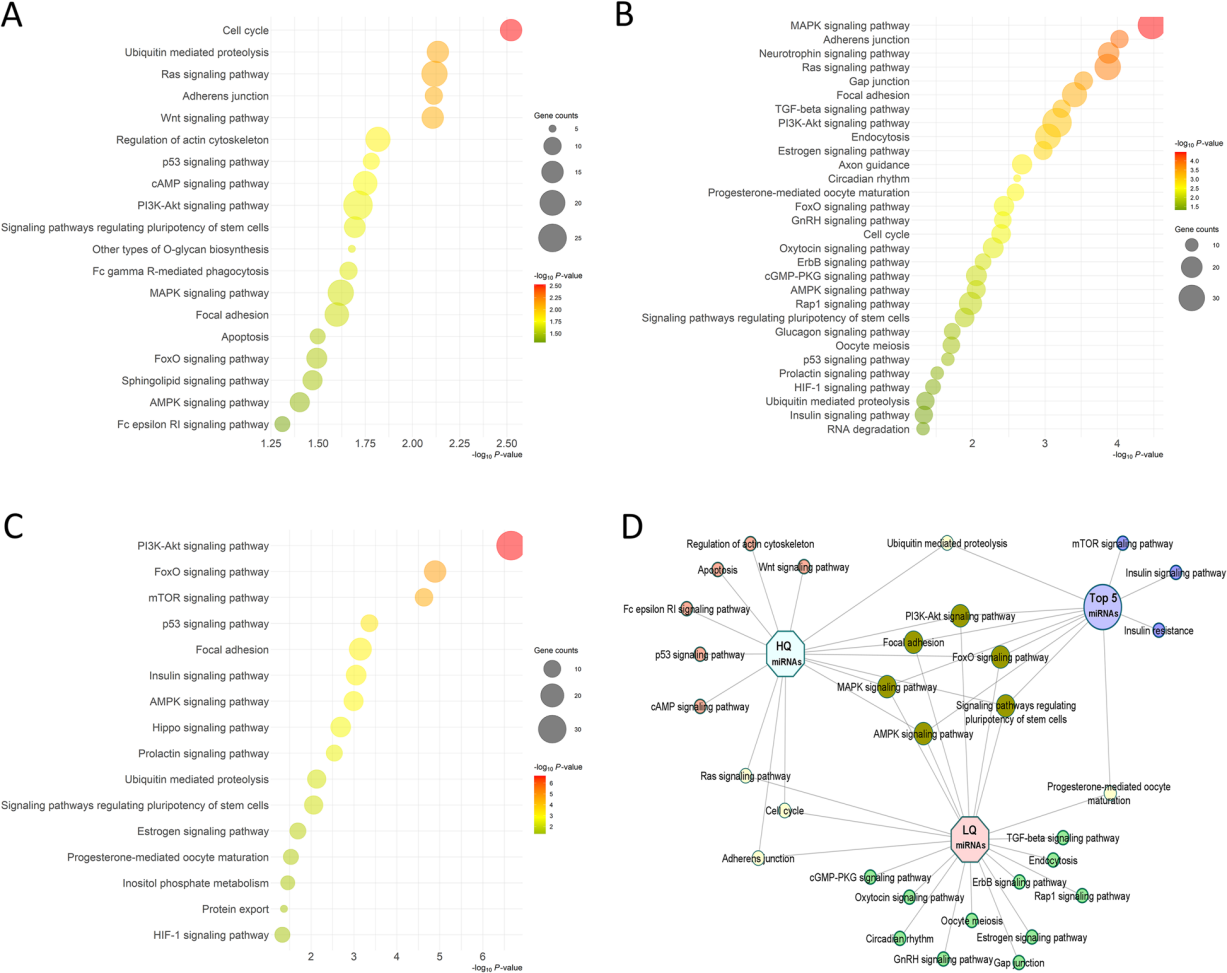
Pathway analysis of differentially expressed miRNA target genes. Bubble plots for the pathways targeted by the elevated miRNAs in the high **(A)** and low quality **(B)** s-EVs groups and by the top five most abundant miRNAs in both groups **(C)**. The color and size of each bubble represent the *P*-value and the number of miRNA target genes in each pathway, respectively. Exclusive and common pathways targeted by elevated miRNAs in the high- (HQ) and low-quality (LQ) s-EVs groups and by the top five most abundant miRNAs in both groups **(D)**

Validated and predicted target gene analysis revealed a total of 860 and 1308 genes are targeted by up- and downregulated miRNAs, respectively, with 193 genes targeted by both. In addition, 702 genes were among the validated and predicted genes and targeted by the top five most abundant miRNAs in both groups. Apoptosis, p53 signaling, and cAMP signaling were among the top pathways targeted by the elevated miRNAs in the HQ group (Fig. 5A, Additional file 1: Table S3). On the other hand, oocyte meiosis, gap junction, TGF-beta signaling, and estrogen signaling were among the top pathways targeted by the elevated miRNAs in the LQ group (Fig. 5B, Additional file 1: Table S3). Signaling pathways including PI3K-Akt, MAPK, AMPK, and FoxO were the top commonly targeted pathways by the elevated miRNAs in the HQ and LQ s-EV groups, as well as by the top five most abundant miRNAs in both groups (Fig. 5C and D, Additional file 1: Table S3). However, genes involved in these common pathways were differentially targeted by each miRNA group, with few genes commonly targeted by two or three miRNA groups, as shown for instance for the PI3K-Akt and MAPK signaling pathways (Additional file 3: Fig. S2).

### Validation of DE-miRNA

**Fig. 6:**
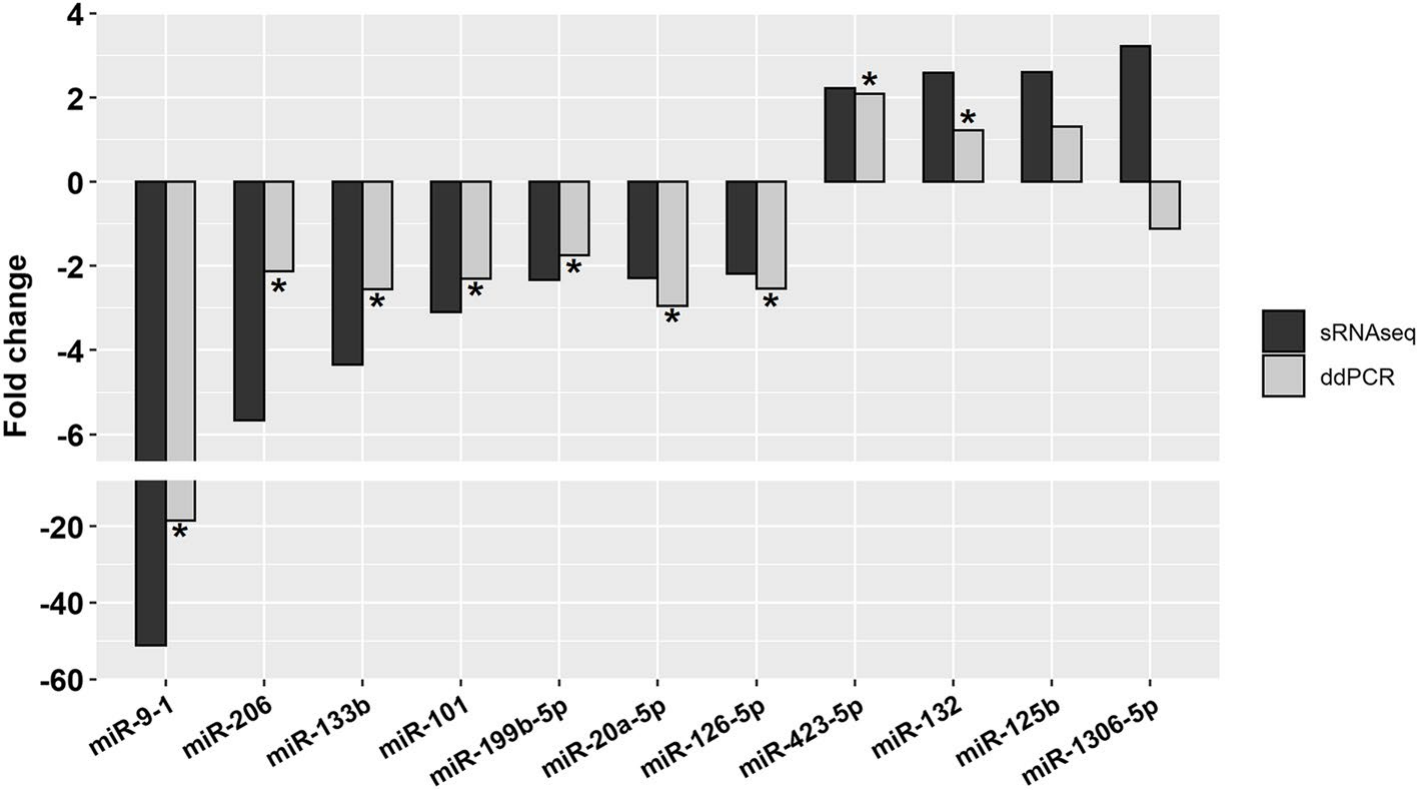
Droplet digital PCR (ddPCR) validation of the selected DE-miRNAs in comparison to the small RNAseq (sRNAseq) analysis. *Statistical significance between the high- (HQ) and low- quality (LQ) s-EVs groups (*P* < 0.05)

A group of 11 DE-miRNAs was selected to validate the miRNA-seq data using ddPCR. All selected miRNAs exhibited the same expression pattern as in the miRNA-seq data (*P* < 0.05) except for two miRNAs (ssc-miR-125b and ssc-miR-1306-5p), which did not differ significantly between the HQ and LQ s-EV groups (Fig. 6).

### Oocyte maturation, developmental competence, and mitochondrial activity after s-EVs co-incubation

**Fig. 7:**
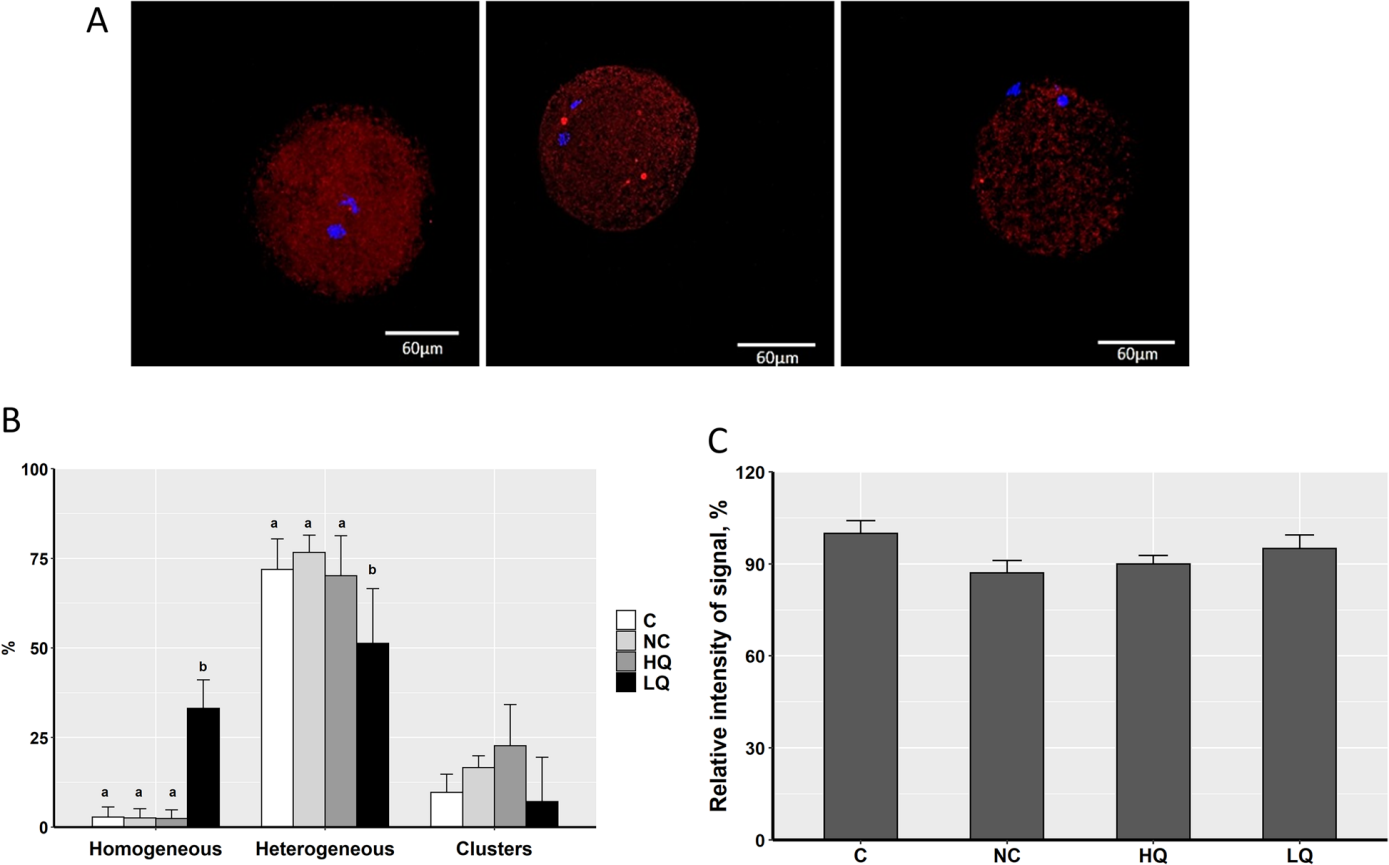
Mitochondrial activity and distribution patterns in porcine MII oocytes. **A** Oocytes were classified into three main distribution patterns: Homogeneous (left), Heterogeneous (middle), or Cluster (right). Scale bar, 60 μm. **B** The proportion of each distribution pattern in porcine MII oocytes after co-incubation with s-EVs of high- or low-quality groups. Bars with different letters indicate significant differences (*P* < 0.05). **C** The relative intensity signal of mitochondrial activity in the different oocyte groups. C: control; NC: negative control; HQ: high-quality; LQ: low-quality

To investigate the effect of s-EVs and their cargos on porcine oocyte maturation and embryonic development, COCs were supplemented with s-EVs of the HQ or LQ groups. We evaluated the nuclear maturation, cleavage, blastocyst rates, and blastocyst cell count in comparison to the non-supplemented control group (C) or PBS-supplemented group (NC). There were no significant differences in nuclear maturation or developmental rates, as well as in the blastocyst cell counts among the experimental groups (Table 3). To examine whether s-EVs might modulate changes in mitochondrial distribution or activity in oocytes after maturation, metaphase II (MII) oocytes were stained with MitoTracker Orange after co-incubation with s-EVs. Based on the mitochondrial distribution patterns, oocytes were categorized into three main categories, homogeneous, heterogeneous, or cluster distribution (Fig. 7A). The results showed that a significantly higher proportion of oocytes co-incubated with the LQ s-EV group was in the homogeneous category and a lower proportion was in the heterogeneous category compared to the other groups (Chi-square test, *N* = 130, *P* < 0.001; Fig. 7B). The overall mitochondrial activity measured as the intensity signal of the stain exhibited no significant differences between the experimental groups (Fig. 7C).

**Table 3:** Maturation and developmental rates of porcine oocytes co-incubated with s-EVs isolated from HQ or LQ FF groups

Group	MII rate % ± SD (n)	Cleavage rate % ± SD (n)	Blastocyst rate % ± SD (n)	Blastocyst cell number ± SD (n)
C	90.07 ± 9.95% (47)	85.34 ± 7.21% (124)	32.40 ± 2.37% (124)	39 ± 5 (27)
NC	85.35 ± 10.95% (55)	86.79 ± 6.17% (125)	29.33 ± 7.93% (125)	39 ± 10 (17)
HQ	75.74 ± 14.29% (53)	76.68 ± 3.83% (129)	25.38 ± 5.13% (129)	42 ± 6 (20)
LQ	87.74 ± 5.75% (55)	75.84 ± 7.98% (154)	25.70 ± 7.71% (154)	45 ± 4 (18)

*C* Control, *NC* negative control, *HQ* high quality, *LQ* low quality

## Discussion

In this study, we identified the miRNA cargo of the s-EVs isolated from porcine FFs corresponding to different oocyte qualities. Furthermore, we investigated the effect of s-EV supplementation during maturation on oocyte developmental competence. We mainly found that s-EV miRNA expression profiles in FFs differed between high- and low-quality corresponding oocytes. These identified miRNAs could be used as non-invasive biomarkers to predict oocyte developmental competence and could provide more insights into the potential role of miRNAs in follicular cell-cell communications and development. Moreover, the supplementation of s-EVs from the FFs of the low-quality group to oocytes during maturation modulates changes in mitochondrial distribution patterns in MII oocytes by increasing the proportion of homogeneous and decreasing the proportion of heterogeneous distribution patterns.

Oocyte quality is one of the key factors that determine the success of IVM, IVF, and the developmental potential of the produced embryo [30]. Several methods are used to evaluate oocyte quality based on morphology, biomarkers expression, and machine learning assistance using oocyte images [31]. Staining GV oocytes with vital stains is another method that can predict oocyte quality and developmental competence. For instance, brilliant cresyl blue (BCB) staining has been widely used to differentiate between growing and fully grown oocytes based on the activity of the G6PDH enzyme [32], since oocytes stained with BCB are more competent than the unstained ones. Another interesting synthetic non-toxic stain that has been used to detect cellular membrane damages is LB stain [33]. It has been used previously for the non-invasive morphological assessment of porcine oocyte quality since it enables the detection of oocytes in the pre-apoptotic stage, expressing high levels of TP53, but still with low levels of pro-apoptotic genes [18]. Moreover, in another study from our group, Bartkova et al. [17] reported LB staining as a non-invasive oocyte selection method that can detect cellular membrane damage in porcine COCs. Although oocyte staining with such stains is considered a non-invasive method, it is still not the optimal approach for oocyte selection, since further treatment and incubation steps need to be incorporated to evaluate the oocytes. Therefore, searching for specific biomarkers in follicular cells or FFs which are associated with oocyte quality could be a much more appropriate strategy for oocyte quality assessment and selection. To avoid any variations in the follicular stages or the physiological conditions of the donors, we collected the FFs from individual healthy ovarian follicles of similar size from prepubertal gilts within similar weight and age ranges. Then, we used LB staining to differentiate between high- and low-quality COCs, and we subsequently collected the corresponding FFs for each category for s-EVs isolation and miRNA identification. We characterized the s-EVs isolated from porcine FFs using three different methods. The size distribution, shape, and the analysis of specific protein markers revealed the successful isolation of s-EVs in agreement with the minimal information for studies of extracellular vesicles (MISEV2018) [34]. Importantly, s-EV samples were negative for CytC and ATP5A proteins as cellular-specific markers. In addition, the RNA electropherogram clearly showed the absence of the ribosomal RNA peaks after total RNA isolation. These verified the isolation procedure and confirmed the degree of purity of the s-EV preparations from other intracellular compartments or cellular contamination [34].

Since the first reported evidence on the EV-mediated transfer of miRNAs between cells [11], EV-miRNAs have been considered to be novel non-invasive molecular markers for the prediction and diagnosis of various pathophysiological conditions [10]. From the FFs, several EV-miRNAs have been reported to be associated with follicular and oocyte development in different mammalian species including porcines [35], humans [36], bovines [37, 38], and equines [39]. However, the mechanism of EV-miRNAs in FFs that influences the oocyte developmental competence remains unclear. In this study, miRNA sequencing analysis identified a total of 42 significantly DE miRNAs between the HQ and LQ s-EV groups. Among the DE miRNAs, the mir-9 family-related miRNAs (miR-9-1 and miR-9) were the most highly elevated miRNAs in the LQ compared to the HQ group, with more than 50-fold miR-9-1 in the LQ group. The miR-9 family was previously known to play roles as repressor mediators of proliferation promoting transcription factors [40] and as tumor suppressors [41]. In humans, miR-9 exhibited a higher expression in the FFs (104 folds) [42] and granulosa cells [43] of women with polycystic ovary syndrome (PCOS) compared to normal women. It has been previously reported that PCOS is highly correlated with poor oocyte quality and subsequently low developmental competence due to consecutive disturbances in the paracrine and/or endocrine follicular microenvironment [44, 45]. Moreover, in our previous study [26] we reported an increase in the expression of miR-9-1 in low-competence porcine oocytes derived from small compared to large follicles. These findings may indicate a possible correlation between the cellular and/or extracellular expression of miR-9 with the oocyte quality. In addition, miR-101 was among the upregulated miRNAs in the LQ compared to the HQ group and was one of the highly abundant DE-miRNAs. Recently, it has been reported that miR-101-3p inhibits goat granulosa cells in vitro proliferation by regulating *CDK4, CCND1, CCNE1,* and *PCNA* expressions and promotes the apoptotic rate by regulating *Bcl-2, Bax, p53,* and *caspase3* expression [46]. The same study reported that, in mouse ovaries, miR-101-3p exhibited unusual ovarian development functions, as reflected by decreased numbers of various follicles as well as small and stunted ovarian fragments. Another interesting group of miRNAs including miR-206, miR-133b, and miR-133a-3p showed a significant up-regulation in the LQ compared to the HQ group. The same three miRNAs exhibited a higher expression pattern in the EVs isolated from the blood plasma of low- compared to high-response heifers to ovarian stimulation [47]. Both miR-206 and miR-133a are highly correlated with E2 deficiency by targeting and reducing the expression of E2 receptor-α, which mediates the biological activity of E2 in ovarian follicular cells and subsequently affects oocyte quality [48–51]. In mice, theca-specific E2 receptor-α knockout leads to a reduction in the oocyte quality and decreased ovulation capacity [52]. Recently, it has been demonstrated that miR-206 decreased the viability and induced apoptosis levels in ovarian granulosa cells by targeting *CCND2* mRNA [53]. Similarly, miR-133a was reported to be a cell proliferation inhibitor and apoptosis promotor in the intestinal epithelial cells [54]. These could explain the higher expression of this group of miRNAs in the FF-EVs of the LQ compared to the corresponding HQ oocytes in this study. On the other hand, a group of 19 miRNAs including miR-132 and miR-320 (one of the highly abundant DE-miRNAs) was significantly up-regulated in the FF-EVs of the corresponding HQ oocytes. Similar to our findings, miR-132 and miR-320 were detected as extracellular miRNAs with a higher expression level in human ovarian FFs of oocytes that yielded top-quality embryos [55]. Moreover, both miRNAs were highly expressed in the ovarian FFs of healthy compared to PCOS patients [56]. In another study, the expression of miR-320 in FFs was positively correlated with human embryonic quality and development. The same study showed that miR-320 knockdown in mouse oocytes strongly decreased their developmental competence [57]. The expression level of miR-132 was higher in the FFs of equine preovulatory compared to dominant follicles, with an indication of the physiological involvement of miR-132 in steroidogenesis, follicle selection, and ovulation [58]. Other interesting miRNAs which were highly abundant in the FF-EVs and exhibited a higher expression level in the HQ compared to the LQ group are miR-125a and miR-125b. Both are members of the mir-10 family and were detected in human FF microvesicles [56]. A recent study demonstrated that miR-125a can be transcribed by all follicular components and play a role in intercellular communication within the follicle [59]. In mice, miR-125a and miR-125b were highly expressed in GV compared to MII oocytes, and it was also found that they play an important role in regulating maternal genes and zygotic genome activation [60].

To investigate the effect of s-EVs on porcine oocyte maturation and embryonic development, we performed a functional experiment by coincubating COCs with s-EVs of the HQ or LQ groups. However, we didn’t observe any significant differences in maturation or embryonic developmental rates after coincubation compared to the control groups. In different mammalian species, several studies have demonstrated that, during in vitro culture, EVs could be uptaken by granulosa or cumulus cells and could be found within the zona pellucida and transzonal projections of cumulus cells [37–39]. Moreover, the uptake of EVs by follicular cells was associated with an increase in endogenous miRNA levels and altered gene expression in in vitro cultured follicular cells [12]. However, the results of studies on the effect of EVs on developmental rates contradict each other. For instance, de Ávila et al. reported that the supplementation of bovine oocytes with FF-isolated EVs does not affect the maturation rate [37]. Moreover, in pigs, it has been suggested that exosomes from FFs are not effective in inducing cumulus cell expansion [61]. In contrast, bovine follicular EVs induced mouse and bovine cumulus cell expansion [38] and improve oocyte competence and survival of heat stress [62]. Several factors including the source of EVs, isolation method, concentration, and incubation time could influence the extent of the EV impact on oocyte or embryonic developmental competence, which could explain these contrary results. For instance, the supplementation of EVs from early antral follicles increased bovine blastocyst rates compared to the control, however, EVs derived from pre-ovulatory follicles exhibited no significant difference [63]. In another study, the supplementation of maturation media with follicular EVs derived by density gradient ultracentrifugation improved the blastocyst rate compared to EVs derived by size-exclusion chromatography [64]. In the same study, EVs derived by both isolation methods improved embryo quality measured as blastocyst cell number and apoptotic cell ratio. More studies considering these factors are needed to optimize the EV co-culture conditions and to determine the mechanisms that regulate oocyte and embryonic development under in vitro conditions. Our results showed that a higher proportion of MII oocytes that were co-cultured with the LQ s-EVs exhibited a homogeneous mitochondrial distribution pattern, and a lower proportion of them was in the heterogeneous pattern compared to the other groups. It is well known that homogeneous and heterogeneous distribution patterns of mitochondria are more commonly observed in GV and MII oocytes, respectively [28]. As oocyte maturation progresses, the mitochondrial distribution changes from homogeneous to a heterogeneous pattern as a sign of cytoplasmic maturation [28, 65]. Several studies reported that released EVs could regulate the function and composition of mitochondria in receptor cells via their metabolite, miRNA, and protein cargos (reviewed in [66]). Not only that, but whole mitochondria or their component parts could be transferred between cells via EVs [67]. This could explain the negative effect of LQ-EVs on the cytoplasmic maturation of MII oocytes by affecting the mitochondrial distribution pattern. However, the precise mechanism by which the EVs could regulate oocyte mitochondrial distribution is still unclear.

## Conclusion

Our results indicated that s-EVs purified from porcine ovarian FFs contain different miRNA cargos that are associated with the quality of the corresponding oocytes. These miRNAs could be used as non-invasive biomarkers for oocyte selection. Moreover, the supplementation of maturation media with s-EVs of the LQ FF group modulates the cytoplasmic maturation of the matured oocytes by affecting the mitochondrial distribution patterns. Further functional studies on the mechanisms by which FF-EVs and their molecular cargos could regulate and maintain oocyte developmental competence will enhance ART outcomes.

## Supplementary Information

**Additional file 1: Table S1.** Summary of sequence reads mapped to the porcine reference genome and annotated against porcine miRNAs listed in the mirBase database. **Table S2.** A complete list of all expressed miRNAs in the high (HQ) and low (LQ) quality s-EVs groups indicated as TMM-adjusted Counts Per Million (CPM). **Table S3.** KEGG pathway analysis for genes targeted by the differentially expressed miRNAs in HQ vs. LQ group and top 5 miRNAs in both groups.

**Additional file 2: Fig. S1.** Mapped reads proportions of small non-coding RNA types in high- (HQ) and low-quality (LQ) s-EVs.

**Additional file 3: Fig. S2.** Interaction networking of genes involved in PI3K-Akt (A) and MAPK signaling (B) pathways and targeted by elevated miRNAs in high- (HQ) and low-quality (LQ) s-EVs groups and by top five most abundant miRNAs in both groups.

## Data Availability

The datasets supporting the conclusions of this article are included within the article and as additional files. The raw FASTQ files and processed CSV files have been deposited in the NCBI’s Gene Expression Omnibus (GEO) with an accession number GSE181182. All relevant data regarding s-EV isolation and characterization were submitted to the EV-TRACK knowledgebase with an EV-TRACK ID EV210251.

## Abbreviations

ARTs: Assisted reproductive technologies
BCB: Brilliant cresyl blue
C: Control group
COCs: Cumulus-oocyte complexes
CPM: Counts per million
ddPCR: Droplet digital PCR
DE: Differentially expressed
EVs: Extracellular vesicles
FC: Fold change
FFs: Follicular fluids
GEO: Gene expression omnibus
HQ: High quality
LB: Lissamine green B stain
LQ: Low quality
MII: Metaphase II
miRNA: MicroRNA
NC: Negative control group (PBS-supplemented group)
NGS: Next generation sequencing
NTA: Nanoparticle tracking analysis
PCA: Principle component analysis
PCOS: Polycystic ovary syndrome
PZM 3: Porcine zygote medium 3
PXM-HEPES: HEPES buffered porcine X medium
SDS: Sodium dodecyl sulphate
sRNAseq: Small RNAseq
s-EV: Small-EV
sncRNA: Small non-coding RNA
TBS: Tris buffered saline
TEM: Transmission electron microscopy
TMM: Trimmed mean of M-values normalization
