# Supplementary figures and images for: Small-extracellular vesicles and their microRNA cargo from porcine follicular fluids: the potential association with oocyte quality
Source: J Anim Sci Biotechnol. 2022 Jun 20;13:82. doi: 10.1186/s40104-022-00723-1 (PMC9208166; doi:10.1186/s40104-022-00723-1)

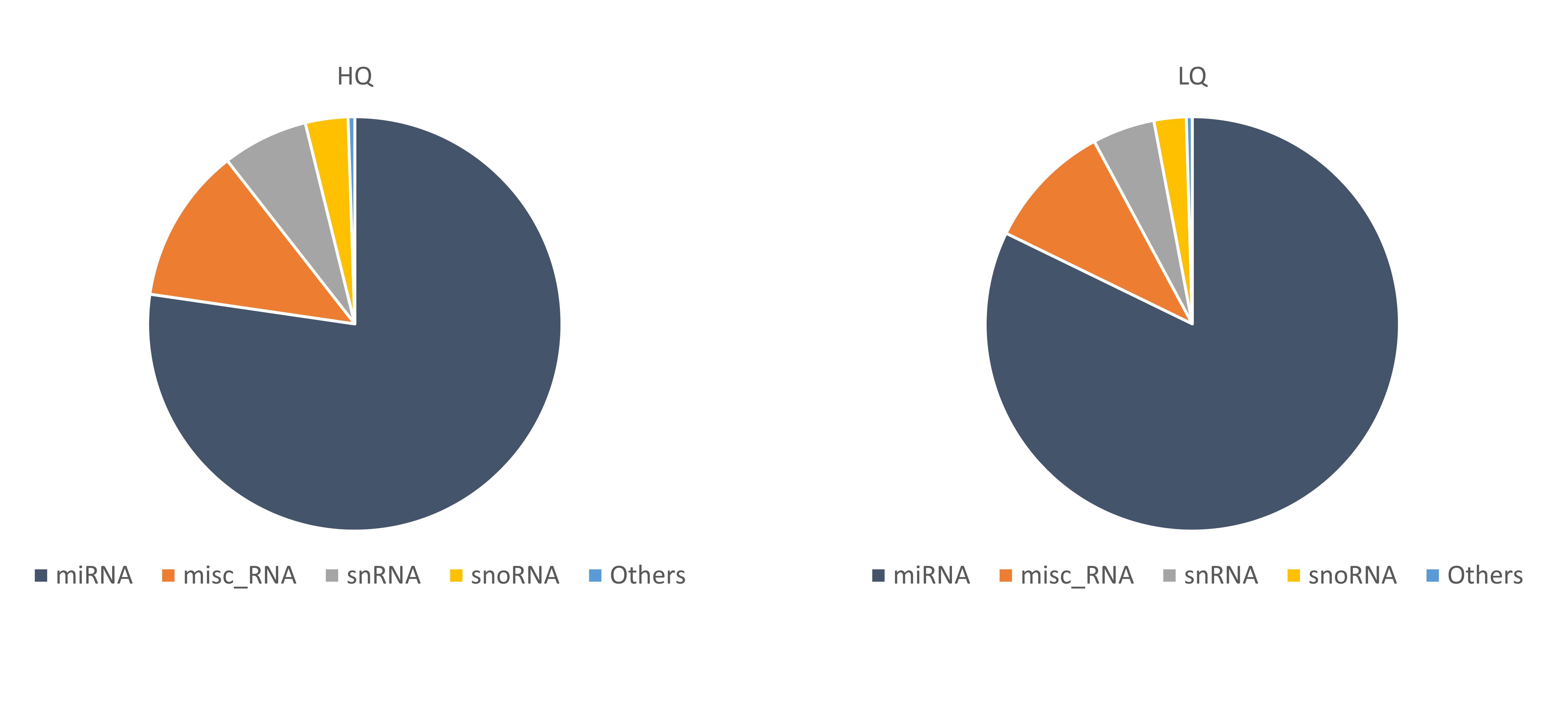

Supplement: Supplementary file 2 — Additional file 2: Fig. S1. Mapped reads proportions of small non-coding RNA types in high- (HQ) and low-quality (LQ) s-EVs. [file 40104_2022_723_MOESM2_ESM.tif]

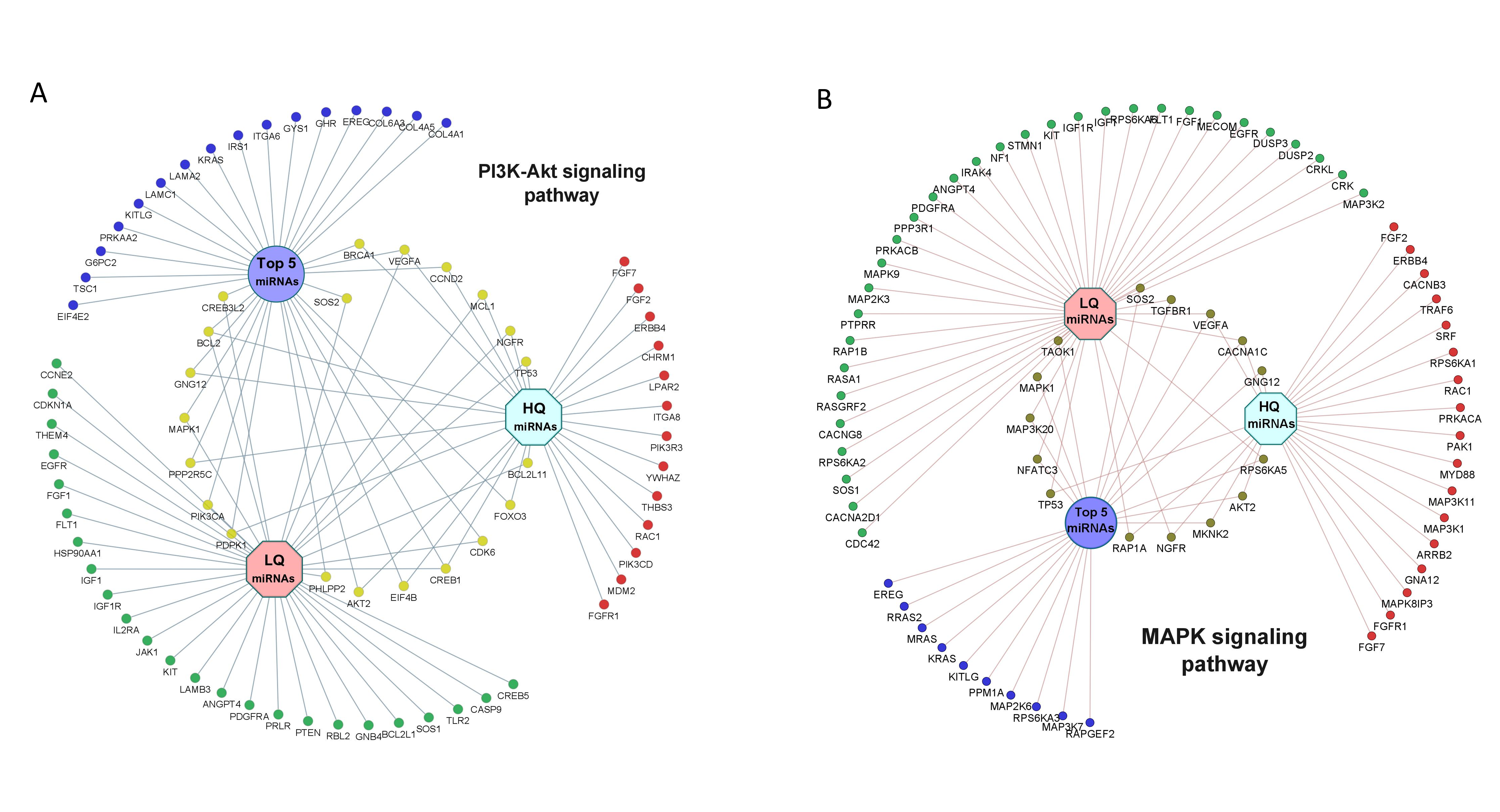

Supplement: Supplementary file 3 — Additional file 3: Fig. S2. Interaction networking of genes involved in PI3K-Akt (A) and MAPK signaling (B) pathways and targeted by elevated miRNAs in high- (HQ) and low-quality (LQ) s-EVs groups and by top five most abundant miRNAs in both groups. [file 40104_2022_723_MOESM3_ESM.tif]
